# Impact of Orthodontic Brackets on the Intraoral Scan Data Accuracy

**DOI:** 10.1155/2016/5075182

**Published:** 2016-11-23

**Authors:** Ji-Man Park, Shin-Ae Choi, Ji-Yun Myung, Youn-Sic Chun, Minji Kim

**Affiliations:** ^1^Department of Prosthodontics, Seoul National University Gwanak Dental Hospital, 1 Gwanak-ro, Gwanak-gu, Seoul 08826, Republic of Korea; ^2^Graduate School of Clinical Dentistry, Ewha Womans University, 911-1 Mok-dong, Yangcheon-gu, Seoul 07985, Republic of Korea; ^3^Department of Orthodontics, Ewha Womans University, 911-1 Mok-dong, Yangcheon-gu, Seoul 07985, Republic of Korea

## Abstract

This study aims to compare the impact of buccal and lingual brackets on the accuracy of dental arch data acquired by 4 different digital intraoral scanners. Two pairs of dental casts, one with buccal brackets and the other with lingual brackets, were used. Digital measurements of the 3D images were compared to the actual measurements of the dental models, which were considered standard values. The horizontal measurements included intercanine widths and intermolar widths. The Mann–Whitney *U* test was performed for comparisons. iTero® and Trios® both showed high accuracy with relatively small maximum deviation of measurements. iTero showed a significantly higher accuracy in most of the arch width measurements on the buccal bracket model than on the lingual model (*P* < 0.05). Zfx IntraScan® and E4D Dentist® produced maximum deviations of more than 2 mm from both the buccal and the lingual bracket models. After comparing the degree of distortion of the arch on the digital scans with actual measurements of the same models, iTero and Trios proved to be excellent in terms of trueness and precision. Nevertheless, digital intraoral scanners should be used more cautiously in arches with lingual brackets than in those with buccal brackets.

## 1. Introduction

Three-dimensional (3D) digital imaging technologies have been utilized in many areas of dental diagnosis and treatment [1–3]. Studies on the accuracy of digital intraoral scanners include those done by Nakamura et al. [4] and Caputi and Varvara [5] and have concentrated mainly on individual prosthesis abutments, rather than the entire arch. Seelbach et al. [6] reported that digital impression-taking systems enabled the fabrication of fixed prostheses with an accuracy level similar to that achieved using traditional impression-taking systems. Ender and Mehl [7] too suggested that digital and conventional impression-taking systems had similar accuracies. However, after comparing images obtained by directly scanning a few teeth with those obtained by scanning a model of the same teeth that had been produced by a conventional impression-taking system, Luthardt et al. [8] reported that the latter approach was more accurate.

In the field of orthodontics, 3D digital scan models can be used not only for diagnostic model analysis, but also for appliances like transfer trays for indirect bracket bonding. Examples of this include Invisalign® (Align Technology, Santa Clara, CA, USA), SureSmile® (Orametrix, Dallas, TX, USA), Incognito™ (3M Unitek TOP Service, Bad Essen, Germany), and Orapix (Orapix, Seoul, Korea). Utilization of 3D digital model systems has helped overcome the disadvantages of plaster casts, such as storage problems, difficulties in data searches, and likelihood of damage, as well as the difficulty and time spent in measurement [9]. Therefore, the reported advantages of the 3D digital models include ease of storage, management and transfer of data, and communication with medical personnel or patients in the dental office, alongside their diagnostic applications [10]. In addition, direct scanning inside the mouth of a patient using an intraoral scanner can reduce the discomfort associated with the use of impression materials. Despite these advantages, surprisingly, the active use of intraoral scanners has mainly increased in prosthodontics and not orthodontics. Since taking impressions during orthodontic treatment can cause a considerable amount of discomfort to the patient, patient convenience can be improved if direct scanning of bracket-bonded arches could produce accurate data. This could prove to be of particular value in cases involving lingual orthodontic treatments, where frequent impression-taking is required and conventional impression-taking is not easy due to the narrow oral structure and tongue. Digital impression-taking can thus be an excellent alternative to conventional methods.

Studies comparing the accuracy of digital scan models with that of plaster casts suggest that 3D digital models have a lower accuracy than plaster casts, albeit in a clinically acceptable range [11–13]. However, very few studies have compared the accuracies of digital scans obtained from arches with bonded buccal and lingual brackets. The aim of this paper is to compare the impact of both buccal and lingual brackets on the accuracy of scan images acquired using 4 different types of digital intraoral scanners.

## 2. Materials and Methods

This study evaluated 4 types of digital intraoral scanners: E4D Dentist (D4D Technologies, Richardson, TX, USA), iTero (1st Generation, Align Technology, Santa Clara, CA, USA), Trios (3Shape, Copenhagen, Denmark), and Zfx IntraScan (Zfx, Dachau, Germany).

**Figure 1:**
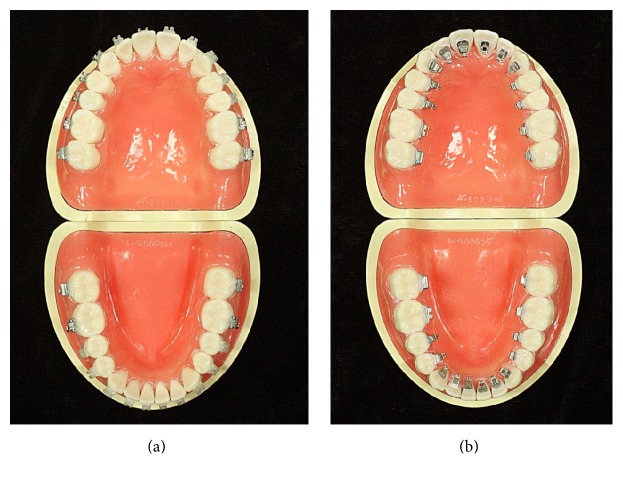
(a) Model B: brackets were bonded on the buccal side of the teeth. (b) Model L: brackets were bonded on the lingual side of the teeth.

Two pairs of identical dental models (085DP-500B.1; Nissin Dental Prod. Inc., Kyoto, Japan) were prepared. Brackets were bonded on the buccal side in one pair (model B) and lingual brackets were bonded on the lingual side in the other (model L) (Figure 1). The brackets used in model B were Victory Series™ MBT 022 (3M Unitek, Monrovia, CA, USA) for the upper right teeth, Clarity™ ADVANCED MBT 022 (3M Unitek, Monrovia, CA, USA) for the upper left teeth, Clarity MBT 022 (3M Unitek, Monrovia, CA, USA) for the lower left teeth, and ODP Lucent™ (Orthodontic Design and Production Inc., Vista, CA, USA) for the lower right teeth. Those used in model L were Ormco 7th Generation™ (Ormco, Orange, CA, USA) for the upper and lower right teeth and STb™ (Ormco, Orange, CA, USA) for the upper and lower left teeth.

A skilled clinician performed the scanning according to the manufacturers' instructions. No powders were applied to the models during scanning with any of the 4 scanners. Each arch was scanned 5 times to obtain a total of 20 pairs of images for the buccal bracket model and the lingual bracket model, respectively.

Actual measurements of the dental models were made using a caliper (700-113 MyCal Lite; Mitutoyo America Corp., Kawasaki, Japan) and were recorded as the standards. The largest and smallest values amongst the 5 repeated measurements were excluded, and the mean of the remaining 3 values was considered as the standard for the digital measurements of the 3D scan images. Digital measurements of the 3D images were made using reverse-engineering software, Rapidform™ 2004 (INUS Technology Inc., Seoul, Korea), once for each image. The intercanine and intermolar widths were measured as the distances between the cusp tips of the right and left canines and mesiobuccal cusp tips of the right and left first molars, respectively.

Mean absolute errors were calculated as the difference between absolute values of digital and actual measurements on 5 repeated images and were used to determine the trueness of the scanner. The maximum deviation was the difference between the maximum and the minimum errors of the 5 repeated images and was considered an indicator of the precision of the scanner.

To compare the 3D bracket images obtained by each scanner with the actual photographs, the bracket on the maxillary right incisor of both models B and L was cropped using the software, from the frontal and lateral view.

### 2.1. Statistical Analysis

Both models B and L were scanned 5 times using 4 scanners, respectively, therefore obtaining five 3D scan images for each arch. To evaluate the errors in accuracy, the mean absolute errors (i.e., the mean of the absolute values of the differences between the digital measured and actual standard values) were calculated along with the maximum deviation, which was the difference between the maximum and the minimum errors of 5 repeated images acquired under identical conditions. The collected data were then analyzed using SPSS Statistics 20.0 (SPSS Inc., Chicago, IL, USA) and the Mann–Whitney *U* test was performed. The confidence interval was 95%.

## 3. Results

**Figure 2:**
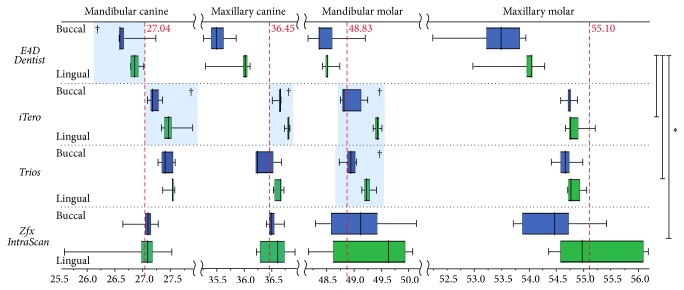
Comparison of intercanine and intermolar widths between model with buccal brackets and model with lingual brackets. The asterisk denotes significant differences between scanners (*P* < 0.05). The dagger denotes significant differences between models with buccal and lingual brackets (*P* < 0.05).

The significantly different measurements between models B and L (*P* < 0.05) were as follows: the mandibular intercanine width measured by E4D Dentist; the maxillary intercanine and mandibular intermolar and intercanine widths measured by iTero; and the mandibular intermolar width measured by Trios (Table 1). iTero showed 3 values that were significantly different between the 2 models, while E4D Dentist and Trios each showed 1. Zfx IntraScan did not show any significantly different measurements between the 2 models. The significantly different measurements indicate that the arch width of model L was larger than that of model B. Thus, the overall horizontal distortion of E4D Dentist was significantly larger than those of the other scanners (Figure 2).

The greatest difference between the maximum and minimum measured values amongst the 10 images of models B and L, which indicates the largest distortion according to buccal and lingual brackets, was 2.47 mm by Zfx IntraScan, followed by 2.06 mm by E4D Dentist, 0.81 mm by iTero, and 0.68 mm by Trios.

The horizontal distortion was assessed by calculating the mean absolute errors between models B and L. The maxillary intercanine and mandibular intermolar and intercanine widths by iTero, as well as the mandibular intermolar width by Trios (*P* < 0.05) (Table 2), were all significantly different. The difference indicated that the mean absolute errors in model B were less than those in model L. Both Zfx IntraScan and E4D Dentist showed 4 mean absolute error values larger than 0.5 mm, while iTero and Trios showed 1 and 0 values, respectively. In terms of maximum deviation, Zfx IntraScan had the highest value (1.44), and this was in the mandibular intercanine width.

**Figure 3:**
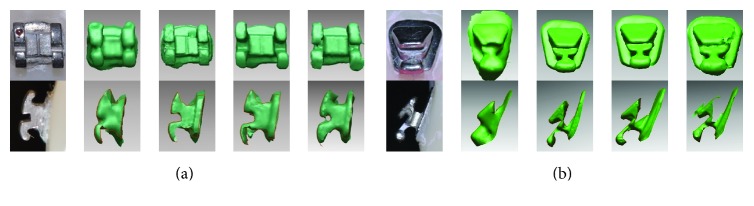
Comparison of images of buccal and lingual brackets. From left to right: real image, E4D Dentist, iTero, Trios, and Zfx IntraScan. (a) Buccal bracket on the right maxillary central incisor. (b) Lingual bracket on the right maxillary central incisor.

Certain features of the dental models were more evident using certain scanner systems, rather than between models B and L. For example, Zfx IntraScan produced unwanted artifacts at the boundary of each bracket, creating irregular border lines. Similarly, borders of the brackets were blurred in images created by E4D Dentist too. In contrast, the images created by iTero and Trios displayed clearer boundaries, with iTero, in particular, producing the sharpest image. iTero also proved capable of reproducing the shape of the slot in its entirety (Figure 3).

## 4. Discussion

The mode of scanning utilized by digital intraoral scanners can be mainly classified into two types. The first involves stitching together a series of pictures to generate a 3D image (stitching-type), and the second involves scanning a surface just as in recording a video (real-time rendering type). Zfx IntraScan system is an example of a real-time rendering system, whereas E4D Dentist, iTero, and Trios are stitching-type systems.

With regard to the intercanine and intermolar width measurements, it was iTero that showed the most significant differences in values between models B and L. Zfx IntraScan did not show any significant differences between the two, possibly because of the large deviations between images repeatedly taken by this scanner (Table 1). Despite being considered to be more accurate since they showed less maximum deviation, the images obtained by iTero and Trios were affected by buccal and lingual brackets, showing larger arch widths in model L (Figure 1). Zfx IntraScan and E4D Dentist showed more than 2 mm of maximum deviations, which could potentially cause significant clinical errors.

The horizontal distortion, assessed by calculating the mean absolute errors between digital and actual measurements, showed significant differences between models B and L with iTero and Trios. Model B showed smaller mean absolute errors, therefore indicating that it produced more accurate images than model L. Compared to iTero and Trios, Zfx IntraScan and E4D Dentist scanners exhibited more mean absolute error values larger than 0.5 mm and therefore can be said to produce less accurate 3D images. Moreover, larger maximum deviations were found in the images produced by Zfx IntraScan and E4D Dentist, indicating that less precise 3D images were produced by these scanners. The mean absolute errors found in this study were 0.09–1.76 mm—a larger range of errors than that observed in a previous study conducted by M.-Y. Lim and S.-H. Lim [13] on plaster casts, which reported a range of only 0.33–1.00 mm. In actual clinical practice, errors more than 0.5 mm cannot be considered negligible, and values such as 1.76, as reported for E4D Dentist, can especially not be overlooked. Other studies of error ranges of arch widths on 3D scan models were done by Han [14], who reported a range of 0.03–0.55 mm, and Park [15], who reported a range of 0.73 mm–0.89 mm.

The maximum deviation indicates the precision of the scanners and was calculated from the maximum and minimum values of measurements of repeated 3D images. Zfx IntraScan and E4D Dentist showed a higher maximum deviation than iTero and Trios, indicating that these two scanners reproduced less consistent images than the former two scanners (Table 2).

The model with lingual bonded brackets was more inaccurate and showed a wider arch width. This may be attributed to the data synthesis process of scanners and the process of merging the pieces of images. In the case of real-time rendering scanners, it is recommended that the overall shape of the arch of the occlusal surface be initially scanned when scanning the full mouth and subsequently the labial and lingual side images be added. When scanning the overall shape of the arch, the anterior region is the most difficult to scan since the incisors are long and labially inclined compared to the posterior teeth. Additionally, the labial aspects of the incisors form undercuts from the occlusal view and therefore are more difficult to scan than the lingual surfaces. As a result, it is highly likely for data errors to instantly occur in this region during the scan. The wider lingual surfaces, and not the incisal edges, of the incisors provide a template for the initial scan, just as the occlusal surfaces do for the posterior teeth. Hence, brackets that are bonded on the lingual surface of the incisors interrupt the initial scanning of the basic arch shape, allowing errors to accumulate early in the process, as more computer calculations are then needed to merge this complicated image data.

In previous studies that evaluated the accuracy of 3D scan images, errors between the 3D images and real models were considered to be a result of either the scanning process, specific algorithms of the software, skill of the clinician, or ability of the scanner to recognize angles, resolution, surface reflectivity, temperature, humidity, methods of digital measurement, or shrinkage of impression materials when using dental casts as a control [16–18]. In our study, however, we have directly measured the models and compared the findings to the 3D images with methods reported to be accurate, therefore ruling out errors due to impression material shrinkage [19, 20].

When examining the features of the brackets on the 3D images, it was found that different scanners produced more differences in images than those between buccal and lingual brackets (Figure 3). The difference in the recording of features by each scanner was more evident in the lingual brackets. Precise scanning is considered difficult in lingual area since interbracket distances are less in lingual brackets than in buccal brackets. The bracket boundaries in iTero and Trios images were relatively clearer in comparison, and, in particular, iTero produced the sharpest images. iTero also reproduced the bracket slot in its entirety.

In this study, dental models were used as the control group. Intraoral scanning in the actual mouth of the patient, however, would not have been any more difficult. A challenge would have presented because of limitations in moving and changing the directions and angles of the intraoral scanner though, since the intraoral environment is restricted and oral structures are located close to each other. While the buccal side allows more space for scanning, the narrow lingual side of the arch, especially in the mandible, can significantly restrict free movement of the scanner. If lingual brackets are bonded under these circumstances, it becomes even more difficult to acquire an accurate 3D image. Therefore, further* in vivo* studies are needed to truly evaluate the accuracy of intraoral scanners.

## 5. Conclusions

This study compared how buccal and lingual brackets may affect the accuracy of 3D images acquired from intraoral scanners. The comparison of horizontal distortions of the arch on 3D models with actual measurements of dental models showed that iTero and Trios were excellent in terms of trueness and precision in these aspects. However, brackets bonded on the lingual side of the teeth reduced the accuracy in arch width measurements. Zfx IntraScan and E4D Dentist displayed maximum deviations of more than 2 mm between models B and L, which are large enough to potentially cause significant clinical errors.

More care must be taken when using intraoral scanners in patients with lingually bonded brackets than in those with buccally bonded brackets. It is important that clinicians select a scanner that can accurately and precisely reproduce images of the dental arch, in order to improve the convenience of treatments.

## Figures and Tables

**Table 1:** Arch width digital measurements on 3D scan model images (unit: mm).

	E4D Dentist				iTero			
	Model B	Model L	Max − min	Significant	Model B	Model L	Max − min	Significant
Maxillary intermolar width	53.49	53.87	2.06	NS	54.75	54.76	0.62	NS
Mandibular intermolar width	48.36	48.52	1.04	NS	48.80	49.43	0.77	*∗*
Maxillary intercanine width	35.49	35.89	0.82	NS	36.66	36.81	0.31	*∗*
Mandibular intercanine width	26.59	26.86	0.66	*∗*	27.18	27.47	0.81	*∗*

	Trios				Zfx IntraScan			
	Model B	Model L	Max − min	Significant	Model B	Model L	Max − min	Significant

Maxillary intermolar width	54.66	54.77	0.64	NS	54.47	54.97	2.47	NS
Mandibular intermolar width	48.94	49.23	0.68	*∗*	49.12	49.63	1.98	NS
Maxillary intercanine width	36.24	36.66	0.50	NS	36.49	36.61	0.70	NS
Mandibular intercanine width	27.41	27.54	0.31	NS	27.10	27.09	1.98	NS

Model B: median measurement of model B.

Model L: median measurement of model L.

Max − min: difference of maximum and minimum measurements among 10 images of buccal and lingual models, which refers to the largest distortion according to buccal and lingual brackets.

^*∗*^ *P* < 0.05 (Mann–Whitney *U* test).

NS: not significant.

**Table 2:** Horizontal distortion of 3D images assessed by mean absolute error in arch widths between digital and actual measurements (unit: mm).

	E4D Dentist			iTero		
	Model B	Model L	Significant	Model B	Model L	Significant
Maxillary intermolar width	1.76 (1.7)	1.23 (1.32)	NS	0.36 (0.31)	0.29 (0.32)	NS
Mandibular intermolar width	0.44 (0.44)	0.29 (0.31)	NS	0.18 (0.39)	0.60 (0.16)	*∗*
Maxillary intercanine width	0.93 (0.59)	0.56 (0.82)	NS	0.18 (0.15)	0.34 (0.10)	*∗*
Mandibular intercanine width	0.39 (0.26)	0.17 (0.24)	NS	0.17 (0.27)	0.49 (0.56)	*∗*

	Trios			Zfx IntraScan		
	Model B	Model L	Significant	Model B	Model L	Significant

Maxillary intermolar width	0.42 (0.58)	0.26 (0.34)	NS	0.79 (1.07)	0.69 (0.95)	NS
Mandibular intermolar width	0.15 (0.11)	0.38 (0.44)	*∗*	0.60 (1.06)	0.81 (1.03)	NS
Maxillary intercanine width	0.20 (0.16)	0.18 (0.16)	NS	0.09 (0.27)	0.26 (0.31)	NS
Mandibular intercanine width	0.35 (0.21)	0.46 (0.22)	NS	0.17 (0.40)	0.45 (1.44)	NS

( ): maximum deviation, which is the difference between maximum and minimum errors of 5 repeated images, which refers to the largest distortion considered as precision of the scanner.

^*∗*^ *P* < 0.05 (Mann–Whitney *U* test).

NS: not significant.
